# Proteome Profile and Quantitative Proteomic Analysis of Buffalo (*Bubalusbubalis*) Follicular Fluid during Follicle Development

**DOI:** 10.3390/ijms17050618

**Published:** 2016-04-29

**Authors:** Qiang Fu, Yulin Huang, Zhiqiang Wang, Fumei Chen, Delun Huang, Yangqing Lu, Xianwei Liang, Ming Zhang

**Affiliations:** 1 State Key Laboratory for Conservation and Utilization of Subtropical Agro-Bioresource, Guangxi University, Nanning 530004, China; gxfuq@163.com (Q.F.); echomide@163.com (Y.H.); zhqwang@gxu.edu.cn (Z.W.); gxchenfumei@163.com (F.C.); delun.huang@uconn.edu (D.H.); luyangqing@126.com (Y.L.); 2 Water Buffalo Institute, Chinese Academy of Agricultural Science, Nanning 530001, China; liangbri@126.com

**Keywords:** buffalo, follicular fluid, proteome, quantitative proteomic, LC-MS/MS

## Abstract

Follicular fluid (FF) accumulates in the antrum of the ovarian follicle and provides the microenvironment for oocyte development. FF plays an important role in follicle growth and oocyte maturation. The FF provides a unique window to investigate the processes occurring during buffalo follicular development. The observed low quality of buffalo oocytes may arise from the poor follicular microenvironment. Investigating proteins found in buffalo FF (BFF) should provide insight into follicular development processes and provide further understanding of intra-follicular maturation and oocytes quality. Here, a proteomic-based approach was used to analyze the proteome of BFF. SDS-PAGE separation combined with mass spectrometry was used to generate the proteomic dataset. In total, 363 proteins were identified and classified by Gene Ontology terms. The proteins were assigned to 153 pathways, including signaling pathways. To evaluate difference in proteins expressed between BFF with different follicle size (small, <4 mm; and large, >8 mm), a quantitative proteomic analysis based on multi-dimensional liquid chromatography pre-fractionation tandem Orbitrap mass spectrometry identification was performed. Eleven differentially expressed proteins (six downregulated and five upregulated in large BFF) were identified and assigned to a variety of functional processes, including serine protease inhibition, oxidation protection and the complement cascade system. Three differentially expressed proteins, Vimentin, Peroxiredoxin-1 and SERPIND1, were verified by Western blotting, consistent with the quantitative proteomics results. Our datasets offers new information about proteins present in BFF and should facilitate the development of new biomarkers. These differentially expressed proteins illuminate the size-dependent protein changes in follicle microenvironment.

## 1. Introduction

Follicles form in the cortex of the mammalian ovary following the recruitment of primordial follicles. During the initial period of ovarian follicle development, a cavity termed the antrum is formed that fills with follicular fluid (FF). FF is composed of secretions from the surrounding granulosa cells (GCs) and selective exudates of blood serum, including proteins, metabolites, ionic compounds and regulatory factors. The blood–follicle barrier plays a central function in determining the composition of FF by regulating the transfer of proteins with a molecular weight <500 kDa [1,2]. FF is an important body fluid involved in reproduction, because it represents the *in vivo* microenvironment for oocyte maturation [3]. Numerous studies have revealed that key substances are essential for the development of oocyte and granulosa cells [4,5,6]. FF also serves as a mediator of communication between the oocyte and GCs. Embryo quality is associated with the presence and concentration of biomarkers in FF [7,8,9]. Thus, understanding the details of FF composition, especially proteins, should provide a comprehensive view of folliculogenesis and aid in the assessment of oocyte quality. Therefore, characterizing the mammalian FF proteome has become an active area of research, especially in the last decade.

Proteomic strategies have been mainly applied to proteome profiling and comparative analysis in protein abundance. Early proteomic research was performed on human follicular fluid (HFF) to compare the complex protein patterns of mature and immature HFF [10]. Subsequently, similar top-down proteomic approaches have been exploited to investigate proteins in FF [11,12,13,14,15]. A bottom-up, high-throughput proteomic approach has been applied to HFF proteomics. Isoelectric focusing and liquid chromatography (LC) were combined to identify 32 proteins [13]. Peptide pre-fractionation and online LC-tandem mass spectrometry (MS/MS) were used to identify the proteome profile of HFF. A total of 246 specific proteins were identified, with the majority of the identified proteins involved in coagulation and immune response pathways [6]. Ambekar *et al.* [16] carried out LC-MS/MS analysis to characterize the proteome of HFF. A total of 480 proteins were identified with high confidence. Proteomic analyses have also been performed in domestic animals: five proteins were identified in bovine FF [17], 113 in mare FF [18], 53 in porcine FF [19] and 21 in canine FF [20]. The use of isobaric tagging for relative and absolute quantitation (iTRAQ) combined with LC-MS/MS approach has become a powerful methodology in proteomic analysis [21]. Many biological processes and pathways associated with *in vitro* fertilization have been investigated by using such methodology [22].

Swamp buffalo (*Bubalusbubalis*) are adapted to hot-humid tropical climate conditions and have low reproductive efficiency. The low quality of buffalo oocytes may be attributable to a poor follicular microenvironment. Currently, no buffalo FF (BFF) proteomics study has been reported. To investigate the protein components in BFF and clarify the variation of size-dependent follicle development, an integrated proteomic study of BFF was performed. First, we used an LC-MS/MS approach to construct a proteome profile of BFF. Second, isobaric and isotopic Tandem Mass Tagging (TMT reagents, similar to iTRAQ reagents) were used to label the BFF proteins according to the size of the follicle, *i.e.*, small (<4 mm) and large (>8 mm). The goal of this research was to reveal any significant expressional changes in proteins during follicular development.

## 2. Results

### 2.1. Proteome Profile of BFF

**Figure 1:**
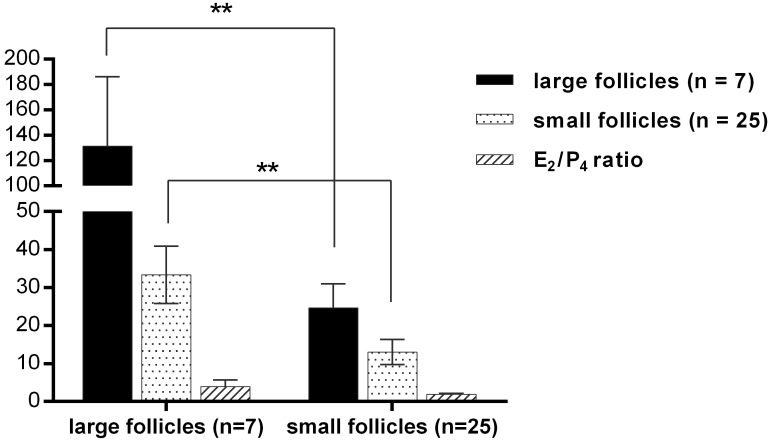
Concentrations of estradiol and progesterone in large and small follicles. The symbol ** indicates significant difference (*p* < 0.01).

Follicles obtained from female buffalo were divided into two groups according to their diameter: (1) small, <4 mm in diameter; and (2) large, >8 mm in diameter. The concentrations of estradiol/progesterone (E_2_/P_4_) in small and large follicles were determined by commercial kits according to the manufacturer’s instructions. Follicles were regarded as pre-ovulatory when the E_2_/P_4_ ratio was >1, and regarded as atretic when the E_2_/P_4_ ratio was <1 (Table 1). One of the large foll icles was atretic follicle (E_2_/P_4_ = 0.63). Concentration of E_2_ (131.41 ± 54.70 ng/mL, *n* = 7) of large follicles was higher than that of small follicles (24.64 ± 6.35 ng/mL, *n* = 25). Concentration of P_4_ (33.35 ± 7.55 ng/mL, *n* = 7) of large follicles was higher than that of small follicles (13.08 ± 3.28 ng/mL, *n* = 25). E_2_/P_4_ ratios demonstrated a significant difference in large and small follicles (large follicles: 3.93 ± 1.72; small follicles: 1.89 ± 0.26). (Figure 1)

**Figure 2:**
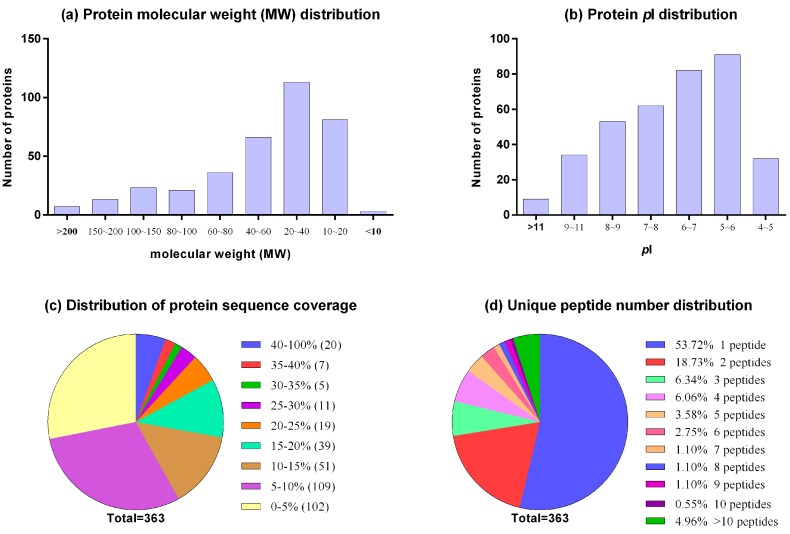
Distribution of the molecular weight, *p*I, sequence coverage and number of unique peptides of the identified proteins in BFF: (**a**) distribution of protein molecular weights; (**b**) distribution of protein *p*I values; (**c**) distribution of protein sequence coverage; and (**d**) unique peptide number distribution.

In proteome profile analysis, proteins derived from two groups of BFF were mixed. They were subjected to SDS-PAGE for pre-fractionation and then analyzed using LC-MS/MS. A total of 74,568 spectra were acquired and matched to 1140 unique peptides. In total, 363 proteins were identified with high confidence. A complete list of the proteins and peptides identified in BFF, with protein scores, Uniprot accession numbers and protein names is presented in Supplementary Table S1. In the distribution of protein molecular weight (*M*_W_) and isoelectric point (*p*I), most *M*_W_s ranged from 10 to 200 kDa (Figure 2a) and the *p*I values ranged from 5 to 10 (Figure 2b). Sequence coverage of one-third of the protein was below 5% (102/363). The average sequence coverage was 12.6% (Figure 2c). All the identified proteins had at least one unique peptide, whereas nearly half the number of identified proteins had more than one and even up to 40 unique peptides (serum albumin, Uniprot accession No. P02769). In total, 46.6% of the proteins (169/363) included at least two unique peptides (Figure 2d). Abundant proteins in BFF were ranked by spectral counts in Supplementary Table S1, which provides a semi-quantitative estimate of the relative protein levels. The most highly abundant protein in BFF was serum albumin (P02769), a known constituent of body fluid. Other abundant proteins included complement C3, apolipoprotein A-I, serotransferrin and Gelsolin, and these proteins have been reported in previously studies.

**Figure 3:**
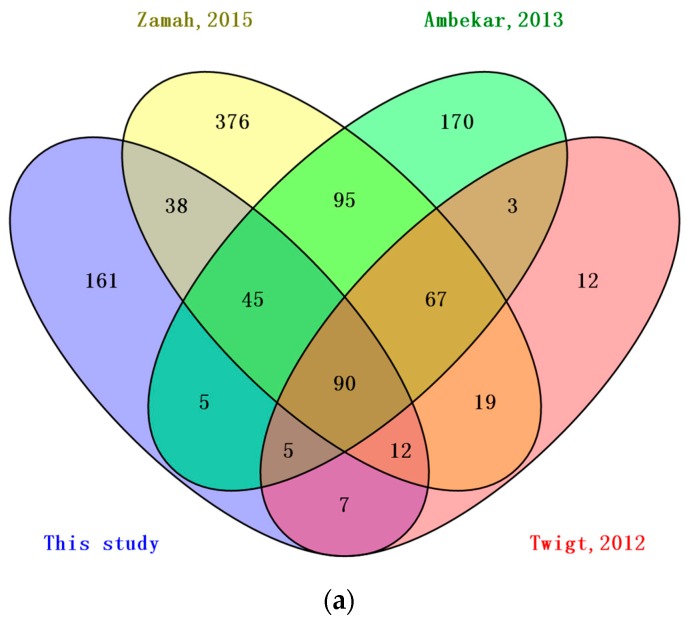

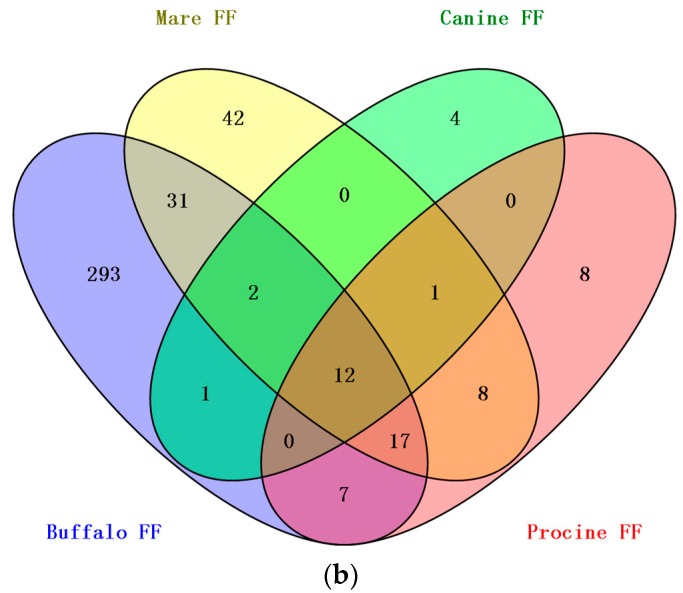
Venn diagram analysis of overlapping proteins: (**a**) overlapping analysis of proteins identified from HFF and BFF; and (**b**) overlapping analysis of proteins identified in the follicular fluid of four mammalian species.

Several studies that have used mass spectrometry to characterize the proteome of human follicular fluid had been reported recently [6,16,23]. The published data were retrieved from these studies and are listed in Supplementary Table S1. The BFF proteome was compared with that of human after conversion of the IDs by homologous BLAST. As shown in Figure 3a, a Venn diagram analysis indicated that 161 proteins were found to be expressed in BFF that have not been reported. Ninety proteins were common to all four studies. By combining our dataset with the other three published studies gave a list of 1105 proteins in the follicular fluid. Furthermore, we compared our results with follicular fluid proteome of the three other mammalian species: mare [18], porcine [24] and canine [20] (the protein symbols are listed in Supplementary Table S2). We found that 12 proteins were simultaneously identified in the four species (Figure 3b) (*i.e.*, albumin, α-2-HS-glycoprotein, apolipoprotein A-I, clusterin, complement C4-A, complement factor B, fibrinogen gamma chain, gelsolin, haptoglobin, retinol-binding protein 4, serotransferrin, and vitamin D-binding protein). This dataset extended the mammalian FF proteome to 416 proteins with an additional 293 high-confidence proteins. The mass spectrometry proteomics data can be fully accessed from the ProteomeXchange Consortium via the PRIDE [25] partner repository.

### 2.2. Gene Ontology (GO) Analysis

**Figure 4:**
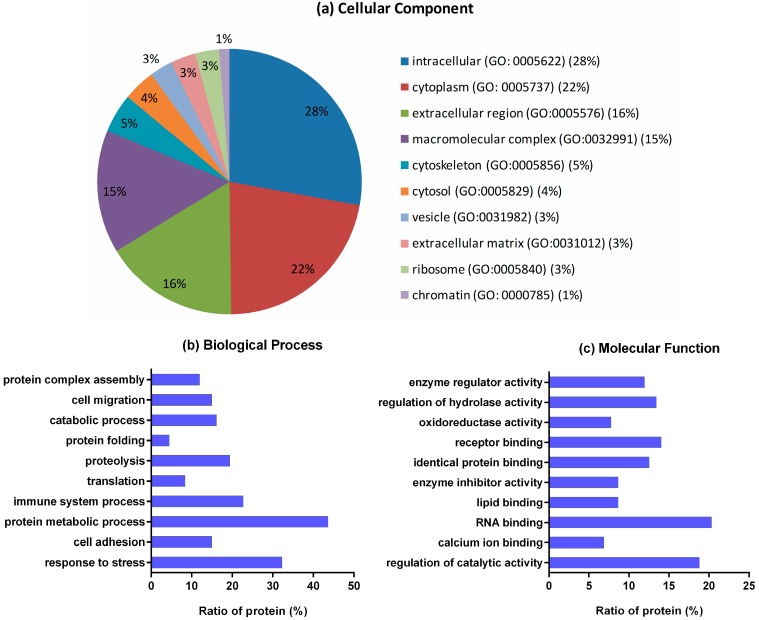
Gene Ontology (GO) analysis of Buffalo follicular fluid (BFF). (**a**) pie chart showing the percentage of cellular component; (**b**) histogram showing the ratio of protein involved in biological process; (**c**) histogram showing the ratio of protein involved in molecular function.

The BFF protein IDs were converted to mouse (*Musmusculus*) IDs of Uniprot database by homologous BLAST. This conversion facilitated analysis by Gene Ontology (GO) annotations, according to cellular component, biological processes and molecular function. The corresponding IDs are presented in Supplementary Table S3. Of the 363 proteins IDs, 335 proteins were annotated in the GO database and only 19 proteins lacked GO annotation information. All GO term annotations are listed in Supplementary Table S3. Some specific GO terms were retrieved and compared. Broadly, the top cellular component categories were “intracellular” (GO: 0005622) (28%) and “cytoplasm” (GO: 0005737) (22%) (Figure 4a). The analysis of biological processes showed that as many as 44% of proteins are involved in protein metabolic process (GO: 0019538). The response to stress and immune system process categories covered 32% and 23% of the identified proteins, respectively, indicating that a significant number of proteins are involved in protecting the follicular microenvironment (Figure 4b). Functional annotation showed that proteins were involved in various molecular functions. As seen in Figure 4c, the majority of the proteins detected were involved in RNA binding (20%) and regulation of catalytic activity (19%). Receptor binding and identical protein binding categories were represented by 14% and 13% of the proteins, respectively.

### 2.3. KEGG Pathway Analysis

To obtain additional pathway information, we further analyzed the identified proteins based on the Kyoto Encyclopedia of Genes and Genomes (KEGG) database. As shown in Table 2, the proteins were assigned to 16 relative pathways (*p* < 0.05). Thirty-three of the proteins belong to the complement and coagulation cascades (mmu04610), which is involved in nonspecific defense against pathogens. The second-largest proportion of proteins (21) was involved in ribosome function (mmu03010). The pentose phosphate pathway (mmu00030), glycolysis/gluconeogenesis (mmu00010), fructose and mannose metabolism (mmu00051) and TCA cycle (mmu00020), four important energy metabolism pathways, were associated with 6, 10, 6 and 4 proteins, respectively. Eight proteins were associated with the HIF-1 signaling pathway. Pathway information and the proteins involved are listed in Supplementary Table S3. Of these, the important pathways are localized and visualized in Supplementary File 1.

### 2.4. TMT-Labeled Quantitative Analysis

To identify protein changes involved in size-dependent follicle development, quantitative analysis of the proteins from large follicles (>8-mm diameter) and small follicles (<4-mm diameter) was performed using TMT 6-plex reagents combined with LC-MS/MS analysis. Proteins isolated from small and large follicles were labeled with mass tags TMT-126 and TMT-129, respectively. Finally, 11 proteins were found to be significantly different (fold-changes of >2 or <0.5) in two experimental replications. The quantitative data of two replications are listed in Supplementary Table S4. Compared with the data derived from the small follicle, six proteins were significantly downregulated and five were upregulated in the large follicle (Table 3). The differentially expressed proteins are involved in various functional processes. The C4A and CFH proteins are associated with the complement coagulation cascade. Upregulation of these proteins indicated that the complement and coagulation cascade is enhanced in large follicles. Four other proteins that were upregulated in large follicles, antithrombin-III, SERPIND1, α-2-macroglobulin and peroxiredoxin-1, are involved in protease inhibition and antioxidant protection. Fructose-bisphosphatealdolase and α-1-acid glycoprotein involved in energy metabolism and transport were upregulated in small follicles. 

### 2.5. Western Blot Analysis of Vimentin

**Figure 5:**
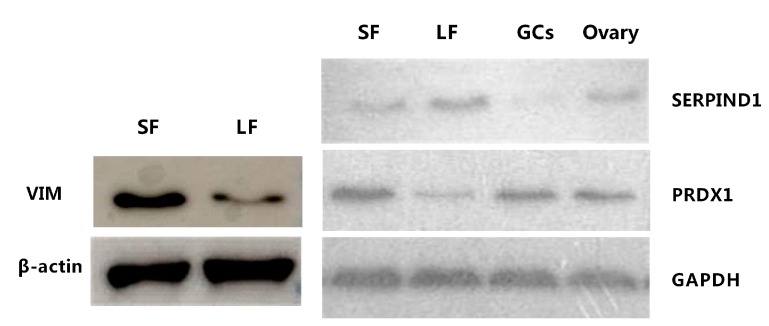
Western blot analysis of the protein expression levels in BFF. SF: <4-mm diameter follicles; LF: >8-mm diameter follicles; GCs: granulosa cells.

Western blot analyses were performed to obtain expression levels of vimentin (VIM), peroxiredoxin-1 (PRDX1) and SERPIND1 proteins in large and small follicles. β-actin and GAPDH were used as the loading control. The β-actin level and GAPDH level had no significant variation in different lanes. Significant changes in signal intensities were observed for VIM and PRDX1, indicating that VIM and PRDX1 expression levels were higher in small follicles than in large follicles. The SERPIND1 expression levels were higher in large follicles than in small follicles (Figure 5). PRDX1 can be detected in granulosa cells (GCs) and ovaries; however, SERPIND1 presents slight expression in GCs and ovaries. The results of Western blotting analysis are consistent with the quantitative proteomic analysis.

## 3. Discussion

Follicular fluid is a complex body fluid that constitutes the microenvironment of follicles, in which the critical events of oocyte and follicle development and cellular communication occur. A thorough identification of the specific components within FF may provide a better understanding of intrafollicular signaling and reveal potential biomarkers of oocyte quality [26]. However, the composition of the BFF proteome remains poorly defined. In our previous study of BFF, traditional two-dimensional electrophoresis (2-DE) combined with mass spectrometry was applied [27]. However, this approach is limited by the poor resolution of 2-DE and was compromised by the large ranges of protein concentrations, spanning ten orders of magnitude. A shotgun proteomic strategy is now the main method of choice for identifying proteins in large-scale proteomic research [28]. Recently, four proteomic studies of HFF using a high-throughput method generated many substantial datasets [6,16,23,29]. In this study, we used an LC-MS/MS strategy to analyze the BFF proteome and identify proteins present in both small and large follicles.

### 3.1. Profile of BFF Proteome

SDS-PAGE pre-fractionation was used and the peptides were subjected to LC-MS/MS analysis. We acquired 130,945 MS spectra and identified 2457 unique peptides that resulted in a total of 363 proteins being identified in BFF. This dataset is the most comprehensive proteomic dataset of mammalian FF reported. However, 78 proteins were assigned to uncharacterized proteins because of incomplete protein annotation of the bovine proteome database. For the convenience of data analysis and mining, the protein list in this study was converted to IDs of human and mouse, which have been deeply annotated. GO analysis revealed that 335/363 proteins (92%) have known GO annotations after ID conversion by homologous BLAST. We carried out subcellular localization and molecular function classification using the DAVID and KOBAS software, respectively. Surprisingly, we found the majority of proteins identified to be intracellular rather than extracellular. We also observed that partial proteins were localized in vesicle, ribosome and cytoskeleton. This finding coincided with the hypothesis that the formation of the follicle cavity may result from the apoptosis of GCs. The presence of intracellular proteins in the FF is likely to be owing to cellular component release from GCs during follicular development [30].

### 3.2. Comparison of BFF, HFF and Plasma

**Figure 6:**
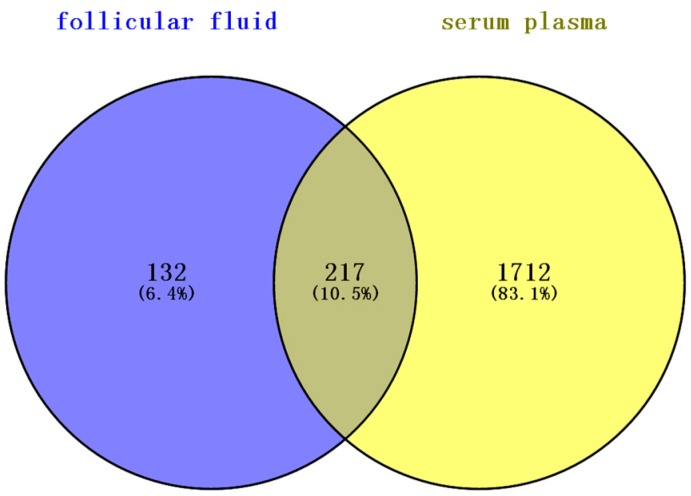
Overlapping analysis of proteins in follicular fluid and serum plasma.

Serum plasma proteins contribute to the composition of all body fluids [31]. During folliculogenesis, follicles become more permeable and this results in higher numbers of serum proteins crossing the blood-follicle barrier [32]. Hence, a comparison of this study to the list of 1929 high-confidence human plasma proteins [33] showed that almost two-thirds (217 proteins) of the total number of proteins identified in this study are shared (Figure 6). This is an expected finding given the serum plasma-filtrate origin of follicular fluid. To investigate the differences between BFF and HFF, we compared the BFF proteins identified with the published HFF proteome reported previously. Half of the proteins (185 proteins) were common to the HFF proteome reported by Zamah *et al.* [23], in which 742 HFF proteins were identified; the largest published HFF. Comparison with the HFF protein reported by Ambekar *et al.* [16] and Twigt *et al.* [6] revealed that 145 proteins and 114 proteins, respectively, were shared between HFF and BFF. FF proteins from four mammalian species, buffalo (this study), mare, porcine and canine, were compared. Only 12 proteins were shared by the four different mammals. This study extended the mammalian FF proteome to 416 proteins with an additional 293 high-confidence distinct proteins.

### 3.3. Protein Functional Classification and Mining

Pathway enrichment showed that 33 proteins are involved in the complement and coagulation cascade system. Some proteins of the complement and coagulation cascade pathway have been reported in previous studies [6,12,13,34]. The complement system and inflammatory processes control follicle wall breakdown during ovulation [35,36]. Complement activation initiates inflammation via recruitment and activation of inflammatory cells. The protein relationship of the complement cascade is illustrated in Supplementary File 1. Folliculogenesis involves various metabolic and proteolytic events that are mediated by a series of enzymes [37]. We have identified several enzymes, including three transferases (GSTA1, GSTM1 and ACAT2) and three hydrolases (UCHL1, PAFAH1B3 and NUDT3). Proteins with enzyme inhibitory function were also detected in this study, e.g., serine protease inhibitor family (SERPIN A1/A3/A5/A10/C1/D1/G1/H1), plasminogen activator inhibitor 1 (SERPIN E1/E2), antiplasmin (SERPIN F1/F2), inter-α-trypsin inhibitor family (ITIH1/H2/H3/H4) and metalloproteinaseinhibitor 2 (TIMP2). SERPINs mediate the activity of proteases and the identification of twelve SERPINs indicates that different SERPINs are involved in follicle development, and may participate in the regulation of follicular extracellular matrix remodeling. We found eight proteins associated with the HIF-1 signaling pathway. HIF-1 is a transcriptional factor that consists of a regulated α subunit and a constitutively expressed β subunit [38]. HIF-1 targets include genes that control cellular growth and metabolism such as enzymes involved in glucose metabolism, and genes that regulate proliferation such as the insulin-like growth factor [39]. In follicle development, Follicle-stimulating hormone (FSH) enhances HIF-1 activity and GCs, and HIF-1 activity is necessary for FSH to induce multiple follicular differentiation markers [40].

### 3.4. Extracellular Matrix (ECM) Proteins and Signaling Proteins

GO analysis and KEGG pathway enrichment revealed that 36 proteins identified (~10%) are involved in the extracellular matrix (ECM), and seven of these proteins are connected to ECM-receptor interaction (mmu04512, Supplementary File 1). ECM proteins are important factors that perform essential functions, including cell signaling, growth, differentiation, maintenance of cell shape and steroidogenesis [41]. Many of these proteins are abundantly secreted by granulosa cells and expressed in cumulus-oocyte complexes (COCs), whose expansion and modulation is essential for follicle growth [42] and ovulation [43]. In particular, several proteins, e.g., heparin sulfate proteoglycan (HSPG2), versican (VCAN) and fibulin-1 (FBLN1), have been reported previously in HFF [16]. We suggest that some of these proteins are critical for oocyte development and fertility.

A complex program of signaling events is required for follicular development and maturation [44]. Multiple proteins associated with signaling were identified, including insulin growth factor (IGF) and IGF binding proteins, grow factor proteins and anti-apoptotic proteins. Metalloproteinase inhibitor 2 (TIMP2) was identified in this study. TIMP2 has been detected and described in GCs of different species previously [45,46]. Cumulus cell apoptosis may be associated with TIMP1. IGF related proteins have been extensively studied in terms of their function on folliculogenesis and steroidogenesis [47]. All proteins identified to be involved in signaling are listed in Table 4. We also found candidate markers associated with oocytes maturation, oocytes quality and abnormal oocytes morphology, as reported in previously [48,49,50,51,52,53] (Table 5).

### 3.5. Protein–Protein Interaction Network

**Figure 7:**
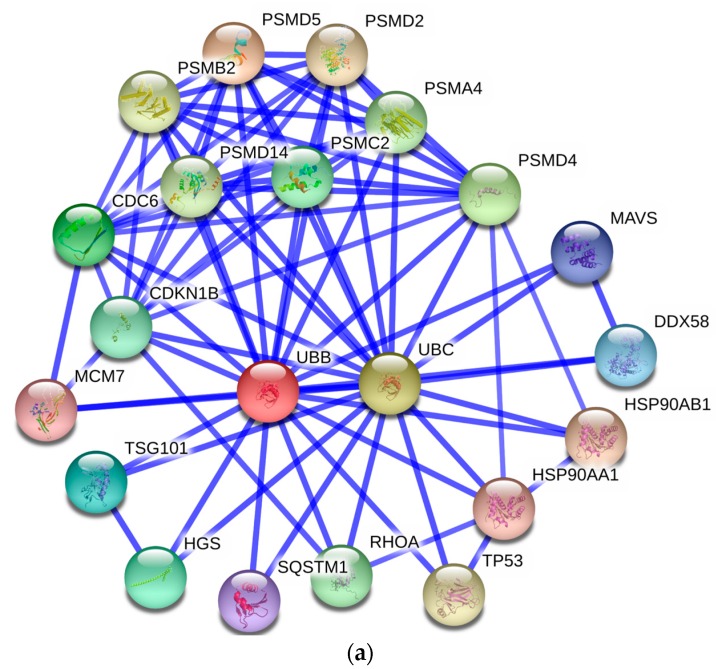

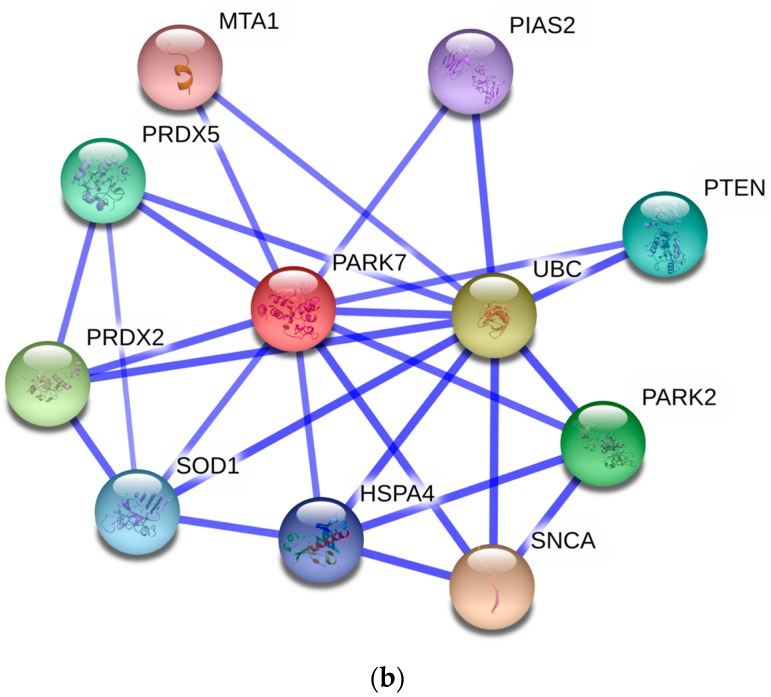
PPI network of ubiquitin B/C and the PARK7 protein: (**a**) the PPI network of ubiquitin B and C proteins; and (**b**) the PPI network of the PARK7 protein.The nodes represent the proteins and the edges represent the corresponding PPI pairs. The thickness of the blue lines represents the confidence score. Required confidence score >0.7.

To provide an efficient way to illustrate the molecular mechanisms of BFF proteins, protein–protein interaction (PPI) information was obtained from the online database of STRING 10. Which is an open source software for predicting and visualizing complex networks [54]. All associations available in the STRING database were provided with a confidence score. Targets with a high confidence score >0.7 were selected to construct the PPI network. The nodes represent proteins and the edges indicate their relations. The largest PPI network included 344 nodes and 792 edges, as shown in supplementary Table S5 and Supplementary File 2. We found more than five clusters in the largest PPI network image, e.g., complement protein family, ribosomal protein family, protease inhibitor, histone protein family and ubiquitin proteins. In particular, ubiquitin B (UBB) and ubiquitin C (UBC) have more than forty edges of interactions. Protein interactions of UBB and UBC were retrieved and are illustrated in Figure 7a. The large number of proteins involved in ubiquitin protein interactions indicates that protein ubiquitination is an active process during folliculogenesis.

Focusing on particular features of identified biological processes, we found two proteins, protein deglycase DJ-1 (PARK7) and chloride intracellular channel protein 4 (CLIC4), involved in both fertilization and reproduction. The PPI networks of PARK7 and CLIC4 were analyzed. As shown in Figure 7b, PARK7 appears to interact with PRDX2, PRDX5 and SOD1, which are involved in intracellular redox signaling. PARK7 plays an important role in cell protection against oxidative stress [55]. We suspect that PARK7 may act as a redox-sensitive chaperone in eliminating peroxides. CLIC4 was found to only interact with ubiquitin C proteins in PPI network. CLIC4 is implicated in diverse cellular processes, ranging from ion channel formation to intracellular membrane remodeling [56]. The function and subcellular targets of CLIC4 remain elusive. Nevertheless, CLIC4 is rapidly recruited to the plasma membrane, suggesting a possible role in ion transport in follicles.

### 3.6. Quantitative Proteomic Analysis

In this work, a TMT labeling strategy was used with the aim of investigating protein variations associated with follicular development. Two experimental replicates were performed to maximize the accuracy of quantitative proteomics (Supplementary Table S5). Only those proteins showing the same trends in expression changes in both replicates were accepted as candidates. Our comparative proteomics analysis showed 11 differentially expressed proteins (>2- or <0.5-fold expression change) between large and small follicles. Of these 11 proteins, six were upregulated and five were downregulated in small follicles.

The ALDOA protein (Uniprot Accession No. A6QLL8) was highly expressed in small follicles, indicating a link to glycolysis. Other research has suggested that glucose metabolism plays an important role in establishing oocyte competence to complete meiosis and fertilization [57,58]. A study on the *ALDOA* gene demonstrated that its expression might play a role in ensuring developmental competence in porcine cumulus–oocyte complexes (COCs) [59]. α-l-acid glycoprotein (ORM1) functions as a transport protein and was highly expressed in small follicles. This protein might facilitate energy transport in an initial stage of follicle growth. Two proteins (complement C4A, complement factor H) belonging to the complement and coagulation cascade system were highly expressed in large follicles. The complement and coagulation cascade systems play roles in maintaining the microenvironment and protection in FF, as in serum [11,12,13,15]. One study showed the involvement of the innate immune function of the complement and coagulation cascade in human FF [34]. Changes in the levels of complement C4A and complement factor H might initiate inflammation via the recruitment and activation of inflammatory cells in FF. Other proteins associated with protease inhibition (*i.e*., SERPINC1 and A2M) were upregulated in large follicles, suggesting that protein degradation was restrained. Furthermore, PRDX1 is involved in redox regulation of the cell and could play a protective antioxidant role during metabolism in large follicles. The expression profiles of PRDX2 in small and large follicles were validated by Western blotting.

An additional finding of this study was the upregulation of VIM in small follicles. VIM, one type of intermediate filament (IF), constitutes part of the cytoskeletal network [60]. The main function of IFs is to maintain a fixed cellular structure. IFs interact with many different proteins, including kinesin-related motor proteins associated with meiosis and mitosis [61] as well as sterol binding proteins, and facilitate lipolysis [62]. Moreover, VIM is involved in the regulation of key signaling pathways that control cell survival, cell growth, cell polarity, intercellular transport and gene regulation [63,64].In this study, the changing VIM levels in BFF provide evidence that IVM might participate infollicular development. Western blot assays confirmed that VIM was highly expressed in small follicles. VIM was reported to be present in plasma [33]. The high level of VIM may be attributable to filtration from blood serum in follicle recruitment.

Overall, this is the first integrated study of BFF proteomics. This database significantly extends the known protein components present in BFF and provides a useful basis for future studies. The discovery of differentially expressed proteins extends number of the possible candidate biomarkers and could help reveal some key biological events in folliculogenesis.

## 4. Materials and Methods

### 4.1. Follicular Fluid Collection and Preparation

Buffalo ovaries were acquired from a local slaughterhouse. BFF was collected from more than ten ovaries in two groups: (1) large BFF from follicles in a dominant stage (>8-mm diameter and showing signs of incipient ovulation); and (2) small BFF from follicles in a less dominant stage (<4-mm diameter). Concentrations of estradiol and progesterone from two groups were determined with 17-βestradiol ELISA kit (Abcam, Cambridge, UK, ab108667) and 17-OH progesterone ELISA kit (Abcam, Cambridge, UK, ab108668) using a microplate spectrophotometer (BioTek epoch, Winooski, VT, USA). The BFF samples from different ovaries were mixed and were centrifuged immediately at 3000× *g* for 20 min to eliminate cells and debris. The supernatants were precipitated by adding six volumes of ice-cold acetone. Proteins were collected by centrifugation at 3000× *g* for 30 min. The precipitates were then resuspended in lysis solution (8 M urea, 4% CHAPS, 50 mM dithiothreitol (DTT) and protease inhibitor, pH 8.0). The protein concentration was determined by 2D Quant kits (GE Healthcare UK Ltd., Little Chalfont, UK) and samples were stored at −80 °C until processed.

### 4.2. SDS-PAGE and LC-MS/MS Analysis

The BFF proteins from large and small follicles were mixed. The mixtures were then boiled for 5 min and subsequently separated on 12.5% polyacrylamide gels and stained with Coomassie brilliant blue R250. The gel lane was cut into eight slices and each slice was cut into fragments for in-gel enzymatic digestion. These were incubated with 50 mM DTT at 55 °C for protein reduction and subsequently incubated with 55 mM iodoacetamide (IAA) at room temperature in the dark for protein alkylation. Samples were digested overnight at 37 °C with trypsin solution (protein/trypsin ratio 50:1 *w*/*w*, pH 8.0). The peptides were extracted from gel pieces twice with 50% acetonitrile (ACN) solution.

**Figure 8:**
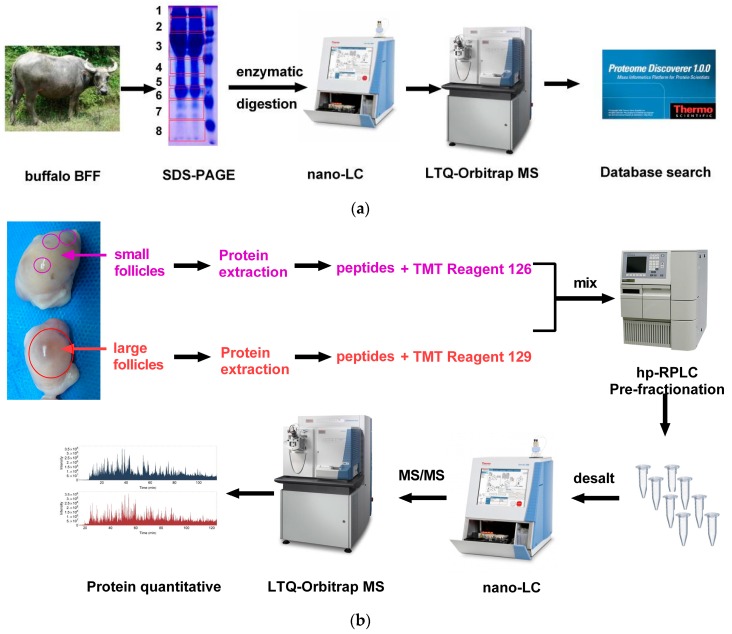
Schematic of proteomic analysis of buffalo follicular fluid (BFF): (**a**) proteomic workflow of BFF protein profile; and (**b**) comparative proteomics workflow for large and small follicles.

The extracted peptides were dissolved in 20 μL of solvent A (2% ACN and 0.1% formic acid (FA)). All eight peptide samples were analyzed online using an LTQ-Orbitrap Elite hybrid mass spectrometer (Thermo Fisher Scientific, Bremen, Germany). LC was carried out using an Easy-nLC 1000 system (Thermo Fisher Scientific, Odense, Denmark) with an Acclaim PepMapnano-trap C18 column (100 μm inner diameter, 2 cm, 5 μm, 100 Å) and an Acclaim PepMap100 C18 column (75 μm inner diameter, 15 cm, 3 μm, 100 Å, both from Thermo Fisher Scientific, Bremen, Germany) at a flow rate of 250 nL/min. The peptides were eluted from the column with a gradient of solvent B (98% ACN, 0.1% FA); 5%–35% solvent B for 45 min, 35%–100% solvent B for 10 min, 100% solvent B for 5 min, total run time 60 min. Eluted peptides were ionized by a Nanospray Flex Ion Source (Thermo Fisher Scientific, Bremen, Germany). Survey scans in the range 150–1800 *m*/*z* were acquired with a MS resolution of 30,000 (at *m*/*z* 400) in the Orbitrap and followed by 10 intensive precursor MS/MS scans by collision-induced dissociation fragmentation at a normalized collision energy of 35% with a MS/MS resolution of 30,000. Dynamic exclusion was enabled with two exclusion counts. The exclusion list size was set to 500 for a duration time of 30 s. Siloxane ions were used for internal calibration (*m*/*z*, 445.1200). The workflow for SDS-PAGE and LC-MS/MS analyses is shown in Figure 8a.

### 4.3. Protein In-Solution Digestion and Peptide Labeling

Equal aliquots of proteins (100 μg each) from large and small follicles were precipitated by ice-cold acetone and resuspended in 100 μL of 100 mM triethylammonium bicarbonate (TEAB). Proteins were reduced by adding 5 μL of 200 mM Tris (2-carboxyethyl) phosphine (TCEP) at 60 °C for 1 h. Cysteine residues were alkylated by adding 5 μL 375 mM IAA for 30 min at room temperature in the dark. The proteins were then digested by adding 2.5 μg trypsin overnight at 37 °C. The peptides were labeled with amine-reactive tandem mass tag reagents (TMT 6-plex Label Reagents Kit; Thermo Fisher Scientific, Rockford, IL, USA, Cat. No. 90064). All the procedures were performed according to the protocol of the manufacturer. Peptides of small follicles were labeled with TMT-126, and the peptides of large follicles were labeled with TMT-129. The two groups of labeled peptides were then pooled and dried in a vacuum concentrator for LC pre-fractionation. The quantitative proteomic workflow is shown in Figure 8b.

### 4.4. Peptide Pre-Fractionation and LC–MS/MS Analysis

The pooled samples were dissolved in buffer A (2% ACN, pH 10.0). Then the samples were pre-fractionated by high-pH reversed-phase liquid chromatography (hp-RPLC) using an XBridge C18 column (4.6 × 250 mm^2^, 5 μm, 130 Å; Waters, Milford, MA, USA) on a Waters e2695 high-performance liquid chromatography system (HPLC, Waters Co., Ltd., Milford, MA, USA). Sixty fractions were collected with a 60 min gradient of 0%–5% buffer B (98% ACN, pH 10.0) for 5 min, 5%–35% buffer B for 45 min and 35%–50% buffer B for 10 min at a flow rate of 0.7 mL/min. The fractions were dried and pooled into eight aliquots. The fractions were desalted using ZipTips C18 Tips (Millipore, Billerica, MA, USA, Cat. No. ZTC18S960) and redissolved in solvent A (2% ACN, 0.1% FA) for LC-MS/MS analysis. The fractionated peptides were used for LC-MS/MS analysis on a nano-LC system (Easy nLC 1000, Thermo Fisher Scientific, Odense, Denmark) combined with an LTQ-Orbitrap Elite mass spectrometer. The parameters for the nano-LC and MS instruments were the same as above. Alternatively, high-energy collision dissociation (HCD) was used for precursor MS/MS fragmentation.

### 4.5. Database Searching and Bioinformatics Analysis

The raw data were processed and searched against the bovine Uniprot protein database (24,213 sequences, released in December 2013; http://www.uniprot.org/taxonomy/9913) using Proteome Discoverer 1.3 (Sequest algorithm, Thermo Fisher Scientific, Bremen, Germany). Parameters were specified as follows: fixed modification = carbamidomethylation (C); variable modification = oxidation (M) and TMT-6plex (Y); one missed trypsin cleavage was allowed; mass tolerance of precursor ions was 20 ppm and that of fragment ions was ±0.8 Da. Only peptides that were filtered with a confidence level of 95% were accepted. The false discovery rate was calculated using peptide validator-based on decoy database searching. Furthermore, GO parameters were analyzed using DAVID 6.7 [65] and KOBAS 2.0 software [66]. The proteins were assigned to the KEGG database for pathway enrichment analysis.

### 4.6. Western Blot Analysis

Variation in differentially expressed proteins in small and large follicles was examined. VIM, PRDX1 and SERPIND1 protein expression levels were determined by a Western blot assay. Twenty micrograms of protein from large and small FF was resolved by SDS-PAGE gel electrophoresis. The VIM antibody was obtained from Abcam (Cambridge, UK; Cat. No. ab8978) and was diluted to 1:3000. The PRDX1 and SERPIND1 antibodies were obtained from Bioss (Beijing, China; Cat. No. bs9842R; bs3875R), which were both diluted to 1:1000. Beta-actin and GAPDH (diluted 1:2000) were detected as the loading control. The proteins were transferred to PVDF membranes with a Hoefer TE22 blotting instrument (Hoefer, Inc., Holliston, MA, USA). The target proteins were detected by chemiluminiscence color or X-ray film. These procedures were done according to published methods [67].

## 5. Conclusions

A total of 363 proteins were identified from BFF using a shotgun proteomic analysis approach. Bioinformatic analysis was employed to better understand the BFF proteome including their classifications by cellular component, biological processes, molecular function and enrichment in KEGG-listed pathways. A quantitative proteomic approach was applied to discover changes in protein levels associated with follicular development. Eleven proteins were differentially expressed between large and small follicles. These proteins are involved in protease inhibition, antioxidant protection and defense responses. Reduced expression of VIM in large follicles compared with small follicles was determined by Western blotting and is consistent with quantitative proteomics.

## Figures and Tables

**Table 1:** Follicles concentrations of estradiol and progesterone and ratios of E_2_/P_4_.

	Large Follicles	Small Follicles
Diameter, mm	>8	<4
Numbers, *n*	*n* = 7	*n* = 25
Estradiol, ng/mL	131.41 ± 54.70	24.64 ± 6.35
Progesterone, ng/mL	33.35 ± 7.55	13.08 ± 3.28
E_2_/P_4_	3.93 ± 1.72	1.89 ± 0.26

**Table 2:** The KEGG pathway analysis of BFF proteins

KEGG ID	Pathway Name	Protein Counts	*p*-Value *
mmu04610	Complement and coagulation cascades	33	8.63 × 10^−27^
mmu03010	Ribosome	21	8.31 × 10^−10^
mmu01200	Carbon metabolism	13	1.08 × 10^−5^
mmu00010	Glycolysis/Gluconeogenesis	10	4.05 × 10^−5^
mmu00030	Pentose phosphate pathway	6	0.00015
mmu01230	Biosynthesis of amino acids	9	0.00028
mmu03050	Proteasome	7	0.00040
mmu00051	Fructose and mannose metabolism	6	0.00176
mmu03040	Spliceosome	10	0.00319
mmu00480	Glutathione metabolism	6	0.00327
mmu00020	Citrate cycle (TCA cycle)	4	0.00699
mmu04612	Antigen processing and presentation	7	0.01812
mmu04512	ECM-receptor interaction	7	0.02425
mmu04810	Regulation of actin cytoskeleton	13	0.02786
mmu04145	Phagosome	10	0.03001
mmu04066	HIF-1 signaling pathway	8	0.04746

* Statistical test method: hypergeometric test/Fisher’s exact test, *p* < 0.05.

**Table 3:** Statistically significant differentially expressed proteins identified from large and small folliclessampled by TMT analysis.

Accession	Protein Name	Gene Symbol	Fold Change *	No. of Unique Peptides	Sequence Coverage %	Functions
Q2KJD0	Tubulin β-5 chain	*TUBB5*	13.69/3.08 Δ	4	11.71	The major constituent of microtubules
A6QLL8	Fructose-bisphosphate aldolase	*ALDOA*	8.22/3.06 Δ	3	8.79	Involved in glycolysis and energy metabolism
Q3SZR3	α-1-acid glycoprotein	*ORM1*	3.06/2.32 Δ	11	2	Functions as a transport protein in the blood
E1BH06	complement C4	*C4A*	0.44/0.36 ∇	10	17.7	Non-enzymatic component of C3 and C5 convertases and thus essential for propagation of the classical complement pathway
F1MSZ6	Antithrombin-III	*SERPINC1*	0.44/0.40 ∇	13	26.9	Most important serine protease inhibitor in plasma that regulates the blood coagulation cascade
Q7SIH1	α-2-macroglobulin	*A2M*	0.31/0.50 ∇	19	14.1	Can inhibit all four classes of proteinases
Q28085	Complement factor H	*CFH*	0.35/0.48 ∇	4	9.55	Functions as a cofactor in the inactivation of C3b by factor I in the alternative complement pathway
P48616	Vimentin	*VIM*	7.78/3.89 Δ	4	7.08	Class III intermediate filament attached to the nucleus, endoplasmic reticulum, and mitochondria
G3N0V0	Uncharacterized protein (fragment)	unknown	4.60/6.29 Δ	7	20.55	Uncharacterized protein
A6QPP2	SERPIND1 protein	*SERPIND1*	0.29/0.44 ∇	6	13.1	Heparin cofactor 2 precursor
Q5E947	Peroxiredoxin-1	*PRDX1*	3.66/3.93 Δ	2	9.55	Involved in redox regulation of the cell. Might play an important role in eliminating peroxides generated during metabolism

* Note: The quantitative proteomic experiments were conducted twice. Protein variations are listed in the fold change column: Δ means the protein was upregulated in small follicles, while ∇ means the protein was upregulated in large follicles.

**Table 4:** BFF proteins with functional roles in signaling.

Uniprot Accession No.	Protein Name	Protein Symbol
Q05717	Insulin-like growth factor-binding protein 5	IGFBP5
F1MUK3	Insulin-like growth factor-binding protein 6	IGFBP6
F1MPP2	Insulin-like growth factor-binding protein 7	IGFBP7
F1MJZ4	Insulin-like growth factor-binding protein complex acid labile subunit	IGFals
F1N430	Metalloproteinase inhibitor 2	TIMP2
P62894	Cytochrome c	CYCS
Q2KIF2	Leucine-rich α-2-glycoprotein 1	LRG1
F1MD95	Calsyntenin-1	CLSTN1
A1A4K5	Ectonucleotidepyrophosphatase/phosphodiesterase family member 2	ENPP2
P13696	Phosphatidylethanolamine-binding protein 1	PEBP1
Q95121	Pigment epithelium-derived factor	SERPINF1

**Table 5:** The biomarkers identified from BFF.

Protein Name	Protein Symbol	Reported Association of Biomarker
Fibronectin (P07589)	FN1	Oocytes maturation [53]
Uncharacterized protein (F1MZX2)	SERPINE2	Positively associated with pregnancy outcome [50]
Superoxide dismutase (A3KLR9)	SOD	Correlated with success of assisted reproductive techniques [51]
Cathepsin B (P07688)	CTSB	Poor quality in cattle oocyte [48]
Polyubiquitin-B (P0CG53)	UBB	Abnormal oocyte morphology; decreased oocyte number; thin zonapellucida [52]
Thrombin (P00735)	F2	Optimal follicular luteinization in mice [49]

## Supplementary Materials

Supplementary materials can be found at http://www.mdpi.com/1422-0067/17/5/618/s1.
