# Supplementary figures and images for: Proteome Profile and Quantitative Proteomic Analysis of Buffalo (Bubalusbubalis) Follicular Fluid during Follicle Development
Source: Int J Mol Sci. 2016 Apr 29;17(5):618. doi: 10.3390/ijms17050618 (PMC4881444; doi:10.3390/ijms17050618)

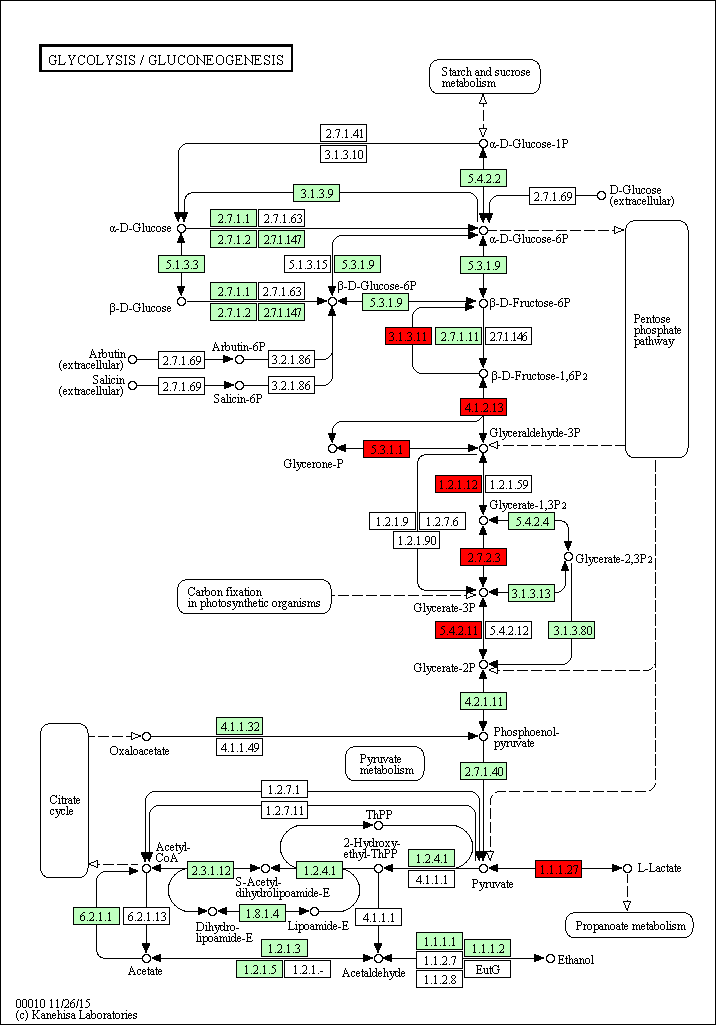

Supplement: Supplementary file 1 [file ijms-17-00618-s001.zip › ijms-111180-Supplementary Materials/supplementary file 1 pathway visualize raw image/mmu00010.png]

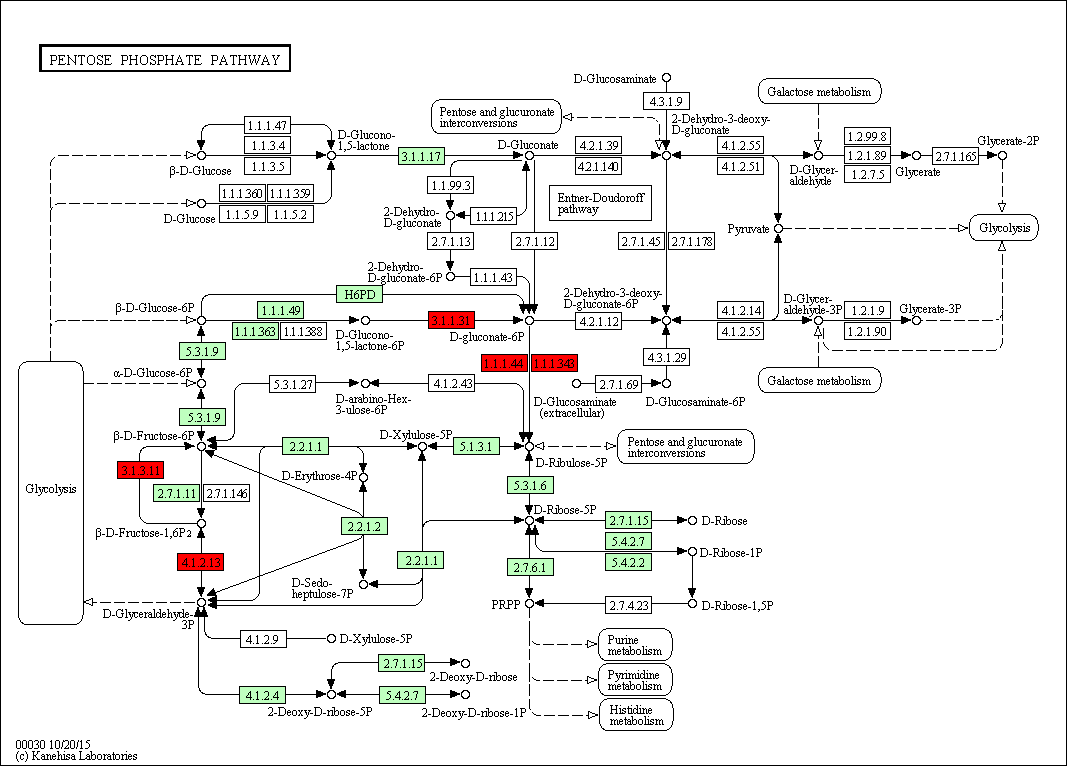

Supplement: Supplementary file 1 [file ijms-17-00618-s001.zip › ijms-111180-Supplementary Materials/supplementary file 1 pathway visualize raw image/mmu00030.png]

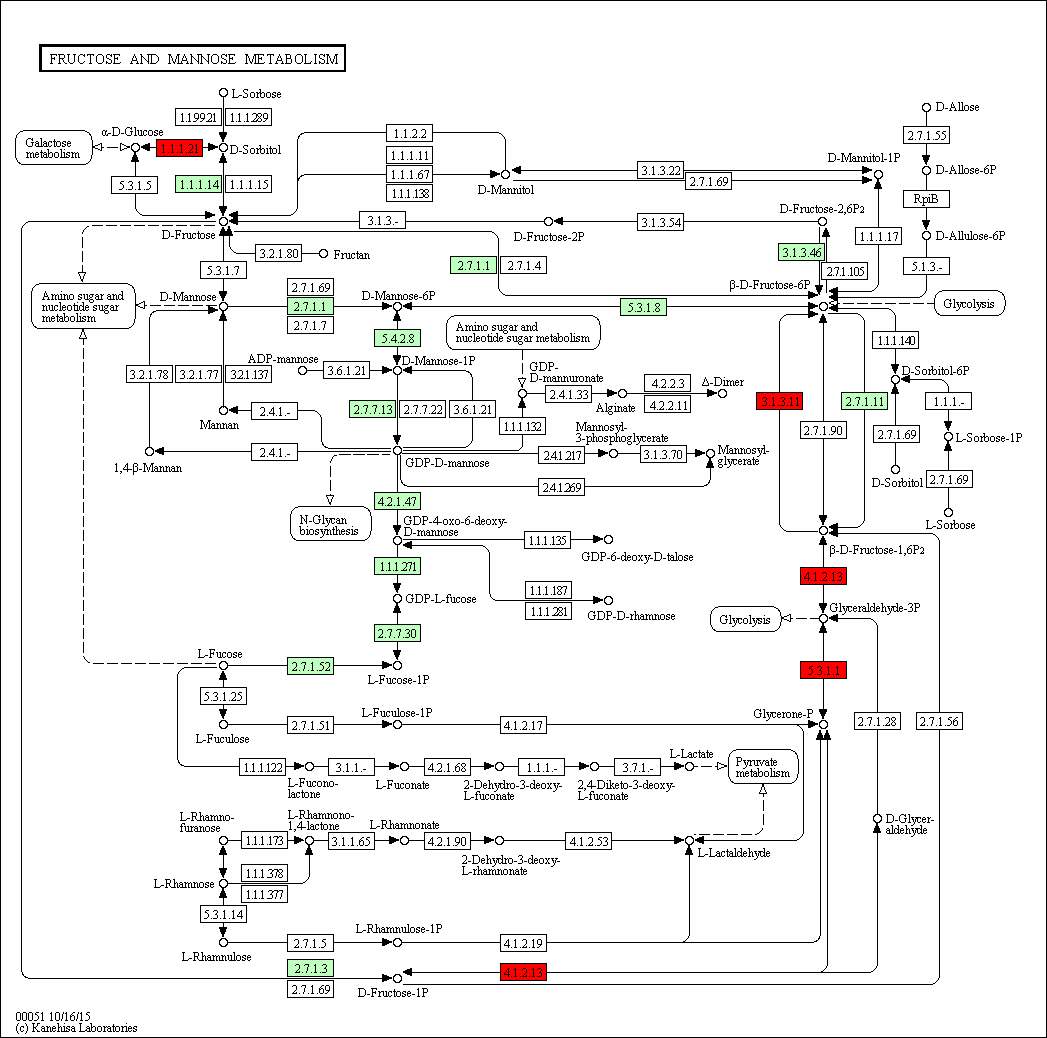

Supplement: Supplementary file 1 [file ijms-17-00618-s001.zip › ijms-111180-Supplementary Materials/supplementary file 1 pathway visualize raw image/mmu00051.png]

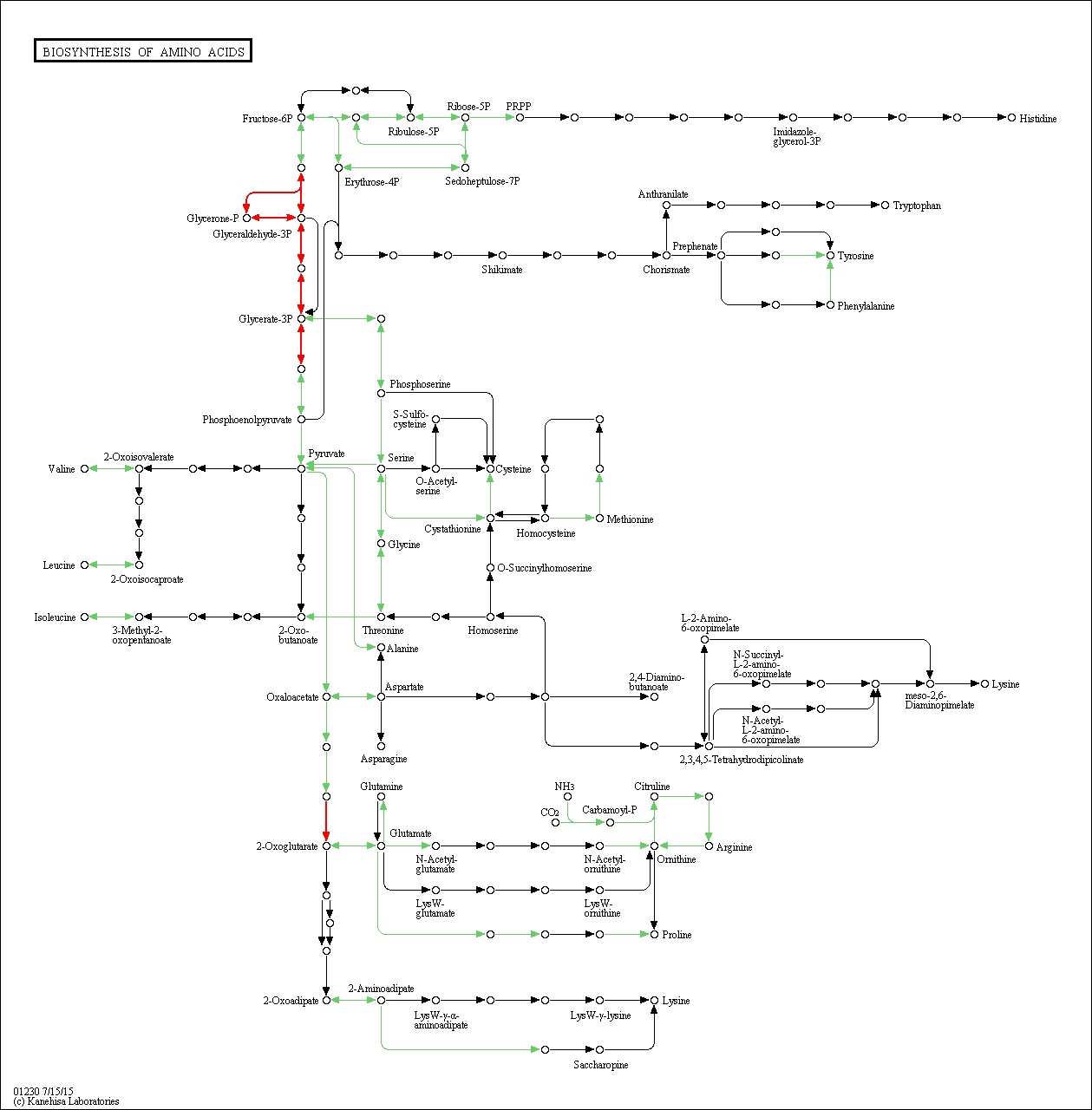

Supplement: Supplementary file 1 [file ijms-17-00618-s001.zip › ijms-111180-Supplementary Materials/supplementary file 1 pathway visualize raw image/mmu01230.png]

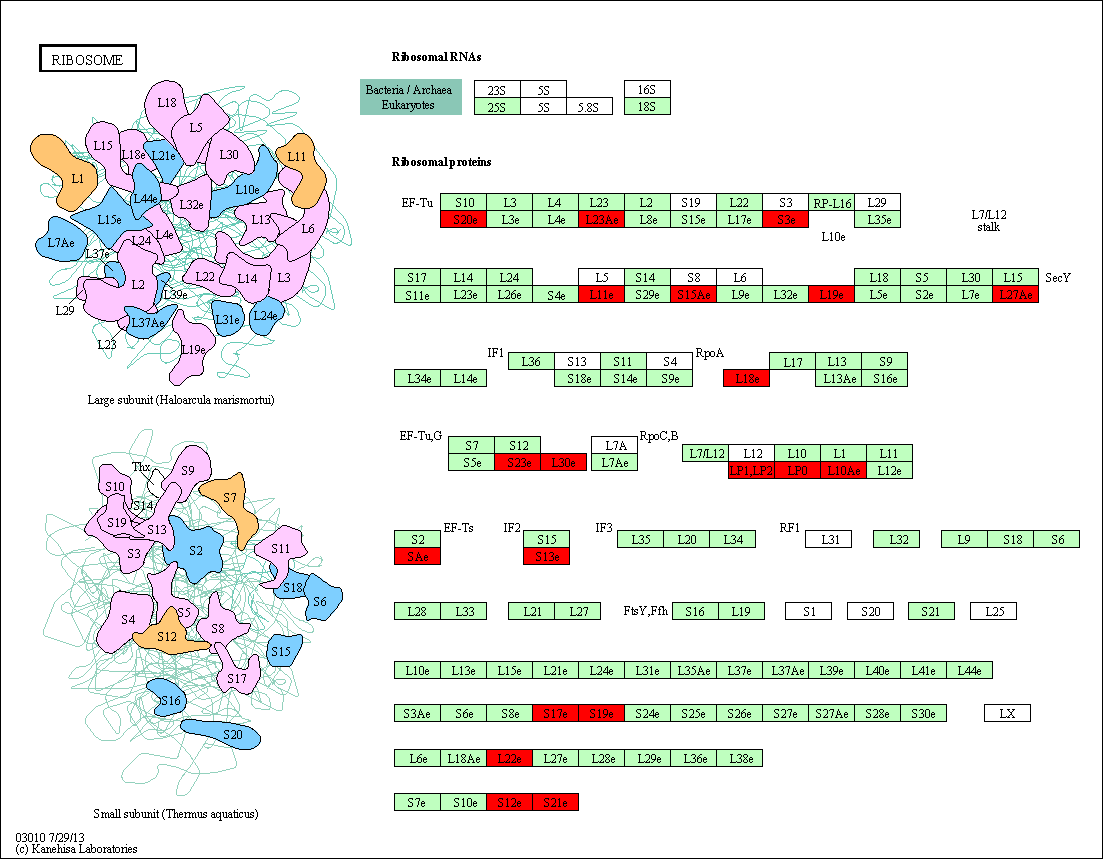

Supplement: Supplementary file 1 [file ijms-17-00618-s001.zip › ijms-111180-Supplementary Materials/supplementary file 1 pathway visualize raw image/mmu03010.png]

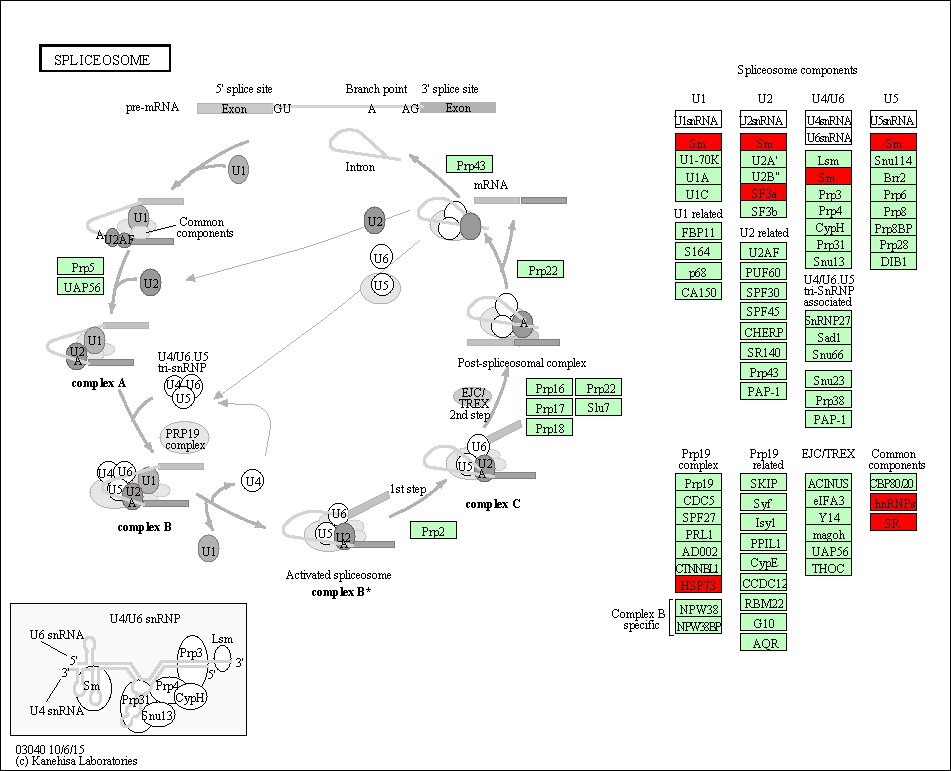

Supplement: Supplementary file 1 [file ijms-17-00618-s001.zip › ijms-111180-Supplementary Materials/supplementary file 1 pathway visualize raw image/mmu03040.png]

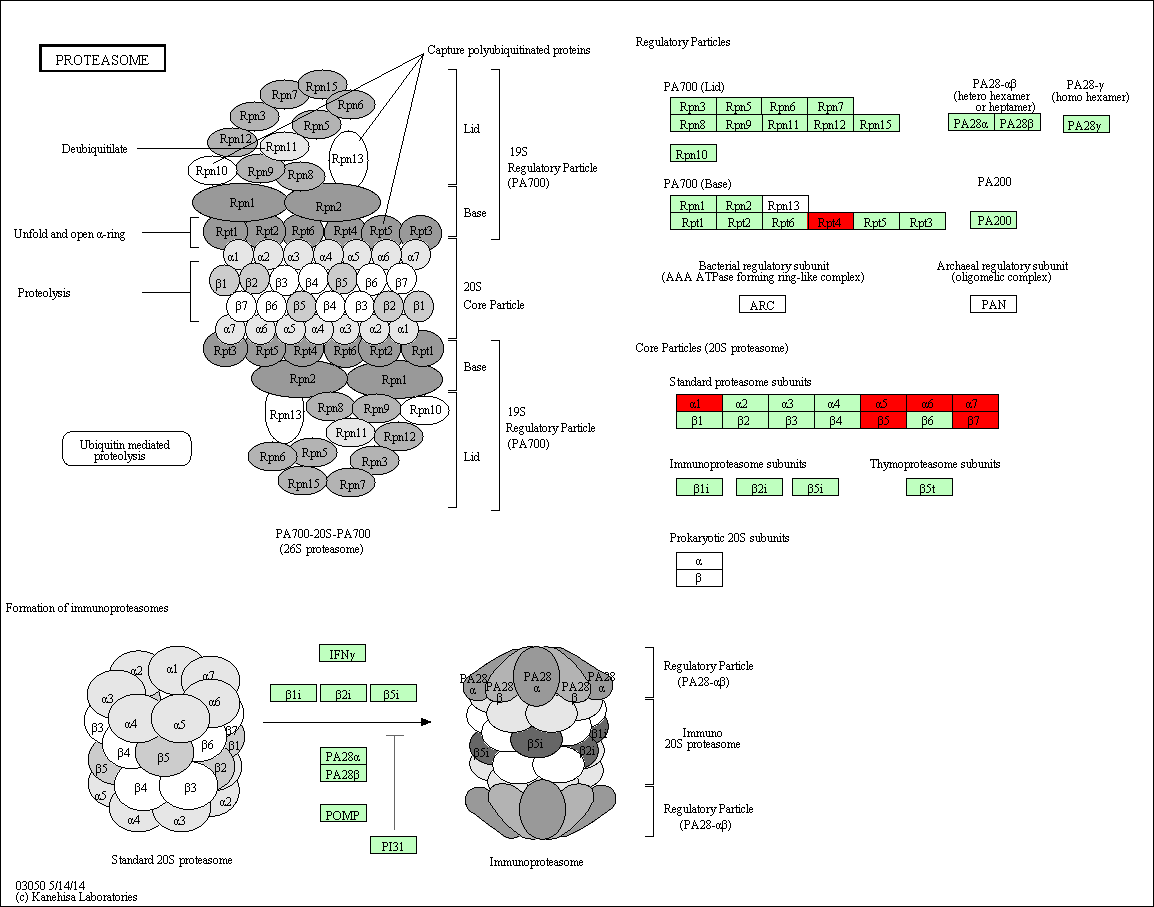

Supplement: Supplementary file 1 [file ijms-17-00618-s001.zip › ijms-111180-Supplementary Materials/supplementary file 1 pathway visualize raw image/mmu03050.png]

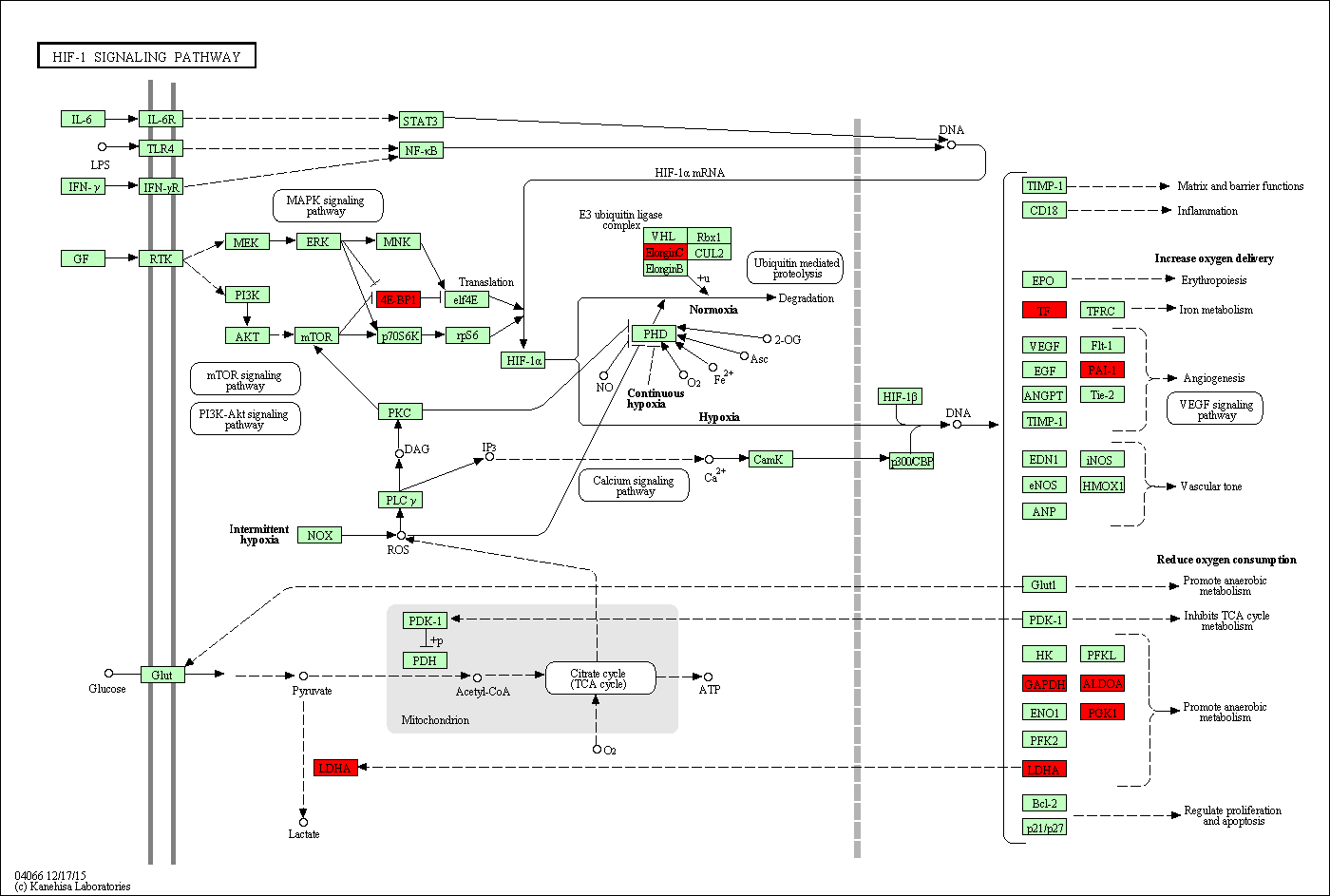

Supplement: Supplementary file 1 [file ijms-17-00618-s001.zip › ijms-111180-Supplementary Materials/supplementary file 1 pathway visualize raw image/mmu04066.png]

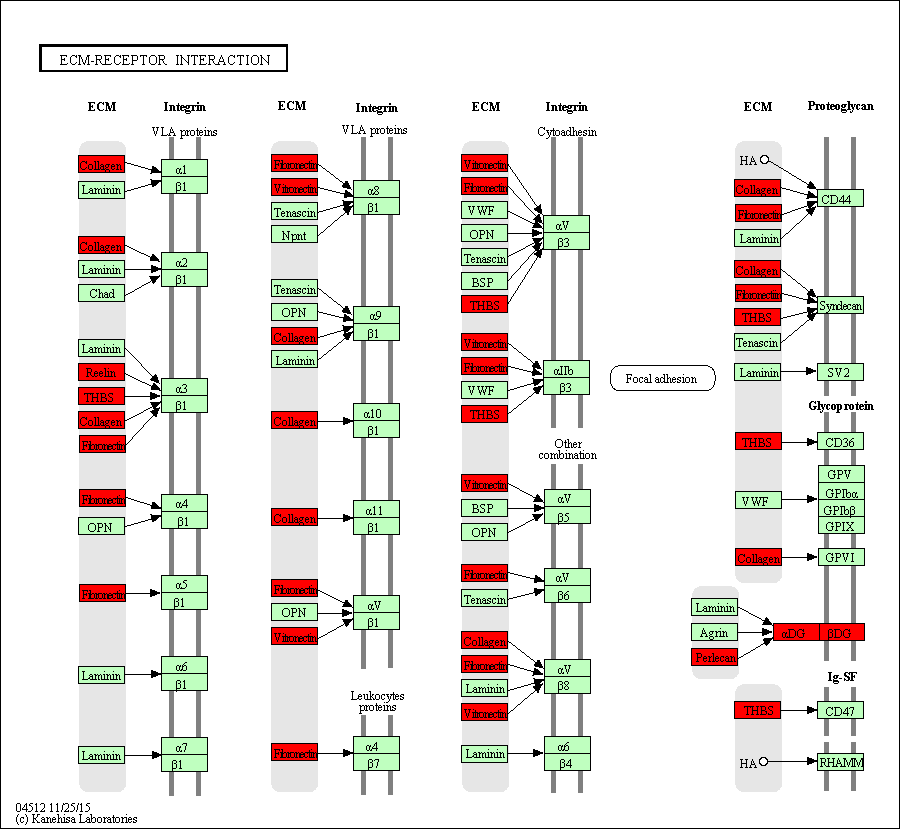

Supplement: Supplementary file 1 [file ijms-17-00618-s001.zip › ijms-111180-Supplementary Materials/supplementary file 1 pathway visualize raw image/mmu04512.png]

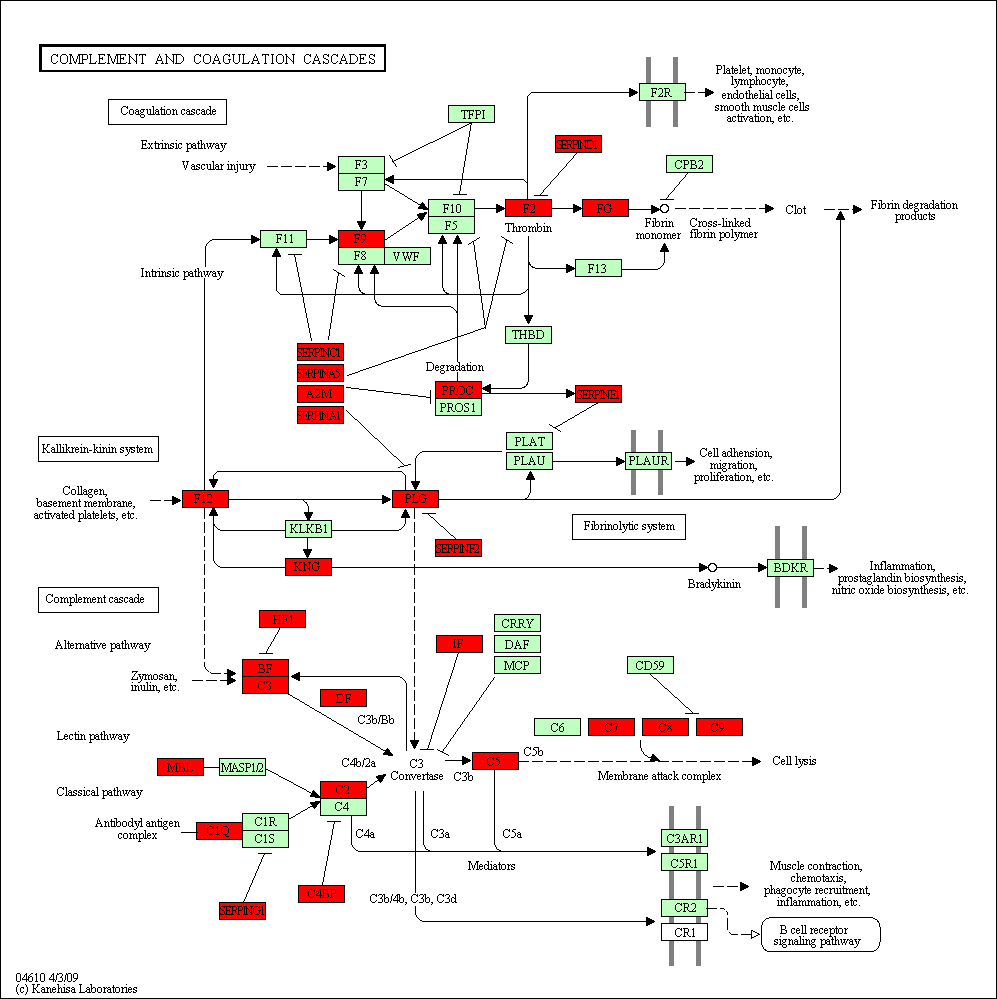

Supplement: Supplementary file 1 [file ijms-17-00618-s001.zip › ijms-111180-Supplementary Materials/supplementary file 1 pathway visualize raw image/mmu04610.png]

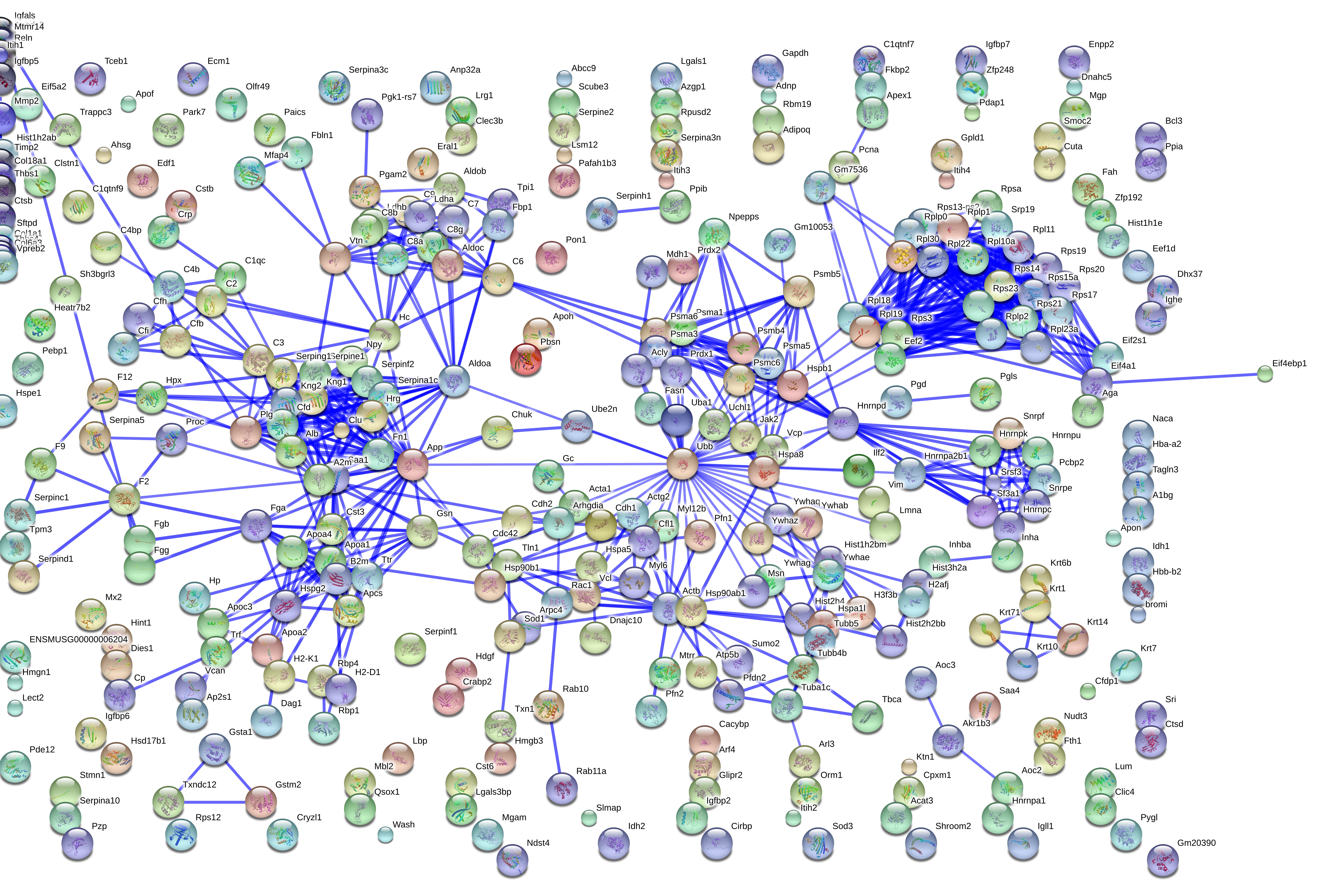

Supplement: Supplementary file 1 [file ijms-17-00618-s001.zip › ijms-111180-Supplementary Materials/supplementary File 2 PPI network.jpg]
